# Maternal depressive symptoms during and after pregnancy are associated with attention-deficit/hyperactivity disorder symptoms in their 3- to 6-year-old children

**DOI:** 10.1371/journal.pone.0190248

**Published:** 2017-12-21

**Authors:** Elina Wolford, Marius Lahti, Soile Tuovinen, Jari Lahti, Jari Lipsanen, Katri Savolainen, Kati Heinonen, Esa Hämäläinen, Eero Kajantie, Anu-Katriina Pesonen, Pia M. Villa, Hannele Laivuori, Rebecca M. Reynolds, Katri Räikkönen

**Affiliations:** 1 Department of Psychology and Logopedics, Faculty of Medicine, University of Helsinki, Helsinki, Finland; 2 University/British Heart Foundation Centre for Cardiovascular Science, Queen’s Medical Research Institute, University of Edinburgh, Edinburgh, United Kingdom; 3 Helsinki Collegium for Advanced Studies, University of Helsinki, Helsinki, Finland; 4 Folkhälsan Research Centre, Helsinki, Finland; 5 Department of Clinical Chemistry, University of Helsinki, Helsinki, Finland; 6 National Institute for Health and Welfare, Helsinki, Finland; 7 Children’s Hospital, Helsinki University Hospital and University of Helsinki, Helsinki, Finland; 8 PEDEGO Research Unit, MRC Oulu, Oulu University Hospital and University of Oulu, Oulu, Finland; 9 Obstetrics and Gynaecology, University of Helsinki and Helsinki University Hospital, Helsinki, Finland; 10 Faculty of Medicine and Life Sciences, University of Tampere, Tampere, Finland; 11 Department of Obstetrics and Gynecology, Tampere University Hospital, Tampere, Finland; 12 Medical and Clinical Genetics, University of Helsinki and Helsinki University Hospital, Helsinki, Finland; 13 Institute for Molecular Medicine Finland, Helsinki Institute of Life Science, University of Helsinki, Helsinki, Finland; Universita Cattolica del Sacro Cuore Sede di Roma, ITALY

## Abstract

Maternal depressive symptoms during pregnancy have been associated with child behavioural symptoms of attention-deficit/hyperactivity disorder (ADHD) in early childhood. However, it remains unclear if depressive symptoms throughout pregnancy are more harmful to the child than depressive symptoms only during certain times, and if maternal depressive symptoms after pregnancy add to or mediate any prenatal effects. 1,779 mother-child dyads participated in the Prediction and Prevention of Pre-eclampsia and Intrauterine Growth Restriction (PREDO) study. Mothers filled in the Center of Epidemiological Studies Depression Scale biweekly from 12+0–13+6 to 38+0–39+6 weeks+days of gestation or delivery, and the Beck Depression Inventory-II and the Conners’ Hyperactivity Index at the child’s age of 3 to 6 years (mean 3.8 years, standard deviation [SD] 0.5). Maternal depressive symptoms were highly stable throughout pregnancy, and children of mothers with consistently high depressive symptoms showed higher average levels (mean difference = 0.46 SD units, 95% Confidence Interval [CI] 0.36, 0.56, *p* < 0.001 compared to the low group), and proportion (32.1% vs. 14.7%) and odds (odds ratio = 2.80, 95% CI 2.20, 3.57, *p* < 0.001) of clinically significant ADHD symptoms. These associations were not explained by the effects of maternal depressive symptoms after pregnancy, which both added to and partially mediated the prenatal effects. Maternal depressive symptoms throughout pregnancy are associated with increased ADHD symptomatology in young children. Maternal depressive symptoms after pregnancy add to, but only partially mediate, the prenatal effects. Preventive interventions suited for the pregnancy period may benefit both maternal and offspring mental health.

## Introduction

Attention-deficit/hyperactivity disorder (ADHD) is characterized by a persistent pattern of inattention, impulsivity, and hyperactivity. It is one of the most prevalent neurodevelopmental disorders in children with prevalence rates varying from 5.9 to 7.1% [1,2]. These symptoms may not only be associated with impairments in academic, socioeconomic and social domains [3], but also predict premature mortality [4].

In the recent decade, the prevalence rates of ADHD have shown a nearly 30% increase [5]. As our genetic makeup has not changed, this increase cannot be attributed to genetic factors. Even though increasing availability of services, recognition of, and screening for ADHD may partly underlie this increase [6], another contributing factor may lie in the exposure to environmental adversities in the prenatal stage of life. Among these adversities are exposure to maternal pre-pregnancy obesity and hypertensive and diabetic pregnancy disorders. They complicate an increasing number of pregnancies and have been linked with an increased risk of ADHD symptomatology in the child [7–10]. They are also among the major underlying causes of preterm birth and low birthweight, which previous studies have also linked with an increased risk of ADHD symptomatology [11–13].

Yet, although extensive research on the effects of maternal depression on offspring outcomes has started to emerge [14,15], relatively little attention has been devoted to the association with offspring ADHD symptomatology. It has been estimated that 7 to 20% of women experience clinically significant levels of depressive symptoms at different stages of pregnancy [16,17]. We are aware of only one large-scale retrospective study which has shown that the mothers of 2-11-year-old children with an ADHD diagnosis were more likely to be diagnosed with depression the year before the birth of the child [18]. Two additional prospective studies have shown that maternal depressive symptoms reported at gestational week 20 were associated with a higher risk of child attention problems at 3 years of age [19], and when reported at gestational weeks 18 and 32, they were associated with a higher risk of child attention and hyperactivity problems at 4 and 11 years of age [19,20].

These studies are, however, limited by a number of reasons. First, they measured depressive symptoms “during the past seven days or last two weeks” at one or two time points during pregnancy not covering the entire pregnancy [19,20]. Second, while two of these studies accounted for maternal depression after pregnancy [19,20], none of the studies tested if maternal depression after pregnancy added to or mediated either fully or partially the prenatal effects. Finally, the studies failed to account for maternal pre-pregnancy obesity and common pregnancy disorders, which in addition to increasing the child’s ADHD risk [8–10], may often also accompany maternal depression [21].

Hence, we tested, in a large sample of pregnant Finnish women, if depressive symptoms, measured biweekly from gestational week 12 onwards until term or delivery, were associated with ADHD symptoms in their 3- to 6-year-old children. The biweekly assessments allowed us to address gestation-week and trimester-specific effects, and maternal re-ratings of depressive symptoms at the time of rating the 3- to 6-year-old child allowed us to address if any effects were specific to the prenatal stage. Our study also tested if maternal depressive symptoms after pregnancy added to or mediated any of the prenatal effects. Finally, we tested if maternal pre-pregnancy obesity, hypertensive pregnancy disorders, and gestational diabetes, or maternal ADHD symptoms accounted for any observed effects. We have previously demonstrated in this cohort associations between maternal depressive symptoms and child internalizing, externalizing, and total problems, including DSM IV-oriented ADHD problems [16] as measured by the Child Behaviour Checklist (CBCL) [22]. This study adds to these findings by testing associations with the Conners’ Hyperactivity Index (CHI) [23], which has been shown to be valid in identifying children 3 years and older with ADHD [23,24]. CHI is a comprehensive measure of ADHD symptoms in children 3 years and older, including aspects of inattention, hyperactivity, and emotional lability [23–25], while the ADHD problems scale of the CBCL is suitable for children 1.5 years and older and focuses more on hyperactive-impulsive and inattentive symptoms [22].

## Materials and methods

### Participants

The Prediction and Prevention of Pre-eclampsia and Intrauterine Growth Restriction (PREDO) study comprises altogether 4,777 mothers and their singleton offspring born alive in Finland between 2006 and 2010 [26]. The women were recruited when they attended the first ultrasound screening between 12+0–13+6 weeks+days of gestation in antenatal clinics at one of the ten study hospitals in Southern and Eastern Finland. Of them, 3,402 (71.2%) assessed their depressive symptoms biweekly during pregnancy.

In 2011–2012 we invited 4,586 mother-child dyads (three children had died before the follow-up, 33 had no data in the Finnish Medical Birth Register (MBR), 55 women declined participation in a follow-up, and for 100 women, addresses were not traceable), and 2,667 (58.2%) participated. Of them 2,312 (68.0% of those with data on depressive symptoms during pregnancy) had pregnancy as well as follow-up data available at the child’s age of 1.9 to 6.3 years (50.6% boys). Since the CHI is validated for children who are 3 years and older [23], we excluded 533 children from the analytic sample. Hence, the current study comprised of 1,779 mother-child pairs who had both pregnancy as well as follow-up data available at the child’s mean age of 3.8 years (Standard Deviation (*SD*) = 0.5 years, range 3.0 to 6.3 years; 51.5% boys).

Compared to the women who were invited but did not participate in the follow-up (*n* = 2,274), the women who participated and whose children in the follow-up were 3 years and older (*n* = 1,779) were older at delivery (31.9 vs. 31.1 years, *p* < 0.001), had more often a tertiary education (61.6% vs. 54.1%, *p* < 0.001), were less often single (1.5% vs. 3.6%, *p* < 0.001), were less often multiparous (57.7% vs. 64.4%, *p* < 0.001), smoked less often throughout pregnancy (2.6% vs. 7.5%, *p* < 0.001), and reported less often a history of a depression diagnosis (9.0% vs. 12.6%, *p* = 0.002).

The PREDO study protocol was approved by the Ethics Committees of Obstetrics and Gynaecology, Children’s Diseases and Psychiatry, and Women, Children and Psychiatry of the Hospital District of Helsinki and Uusimaa and by the participating hospitals. All participating women signed informed consent forms and all procedures contributing to this work comply with the Helsinki Declaration of 1975, as revised by the 59th WMA General Assembly, Seoul, Republic of Korea, October 2008.

### Child ADHD symptoms

At the child’s age of 3 to 6 years, their mothers rated the ten CHI questions on the child’s behavioural symptoms of ADHD on a scale of “not at all” (0) to “very much” (3) [23]. A sum-score of 10 or above indicates clinically significant ADHD symptoms [27]. The scale has good internal consistency [24,25] and discriminant validity [24,25]. In our sample, it showed high internal consistency (α = .91).

### Maternal depressive symptoms during and after pregnancy

Depressive symptoms were reported biweekly up to 14 times throughout pregnancy starting from 12+0–13+6 to 38+0–39+6 weeks+days gestation or delivery using the Center for Epidemiological Studies Depression Scale (CES-D) [28]. The CES-D has 20 questions rated on a scale of none of the time (0) to all the time (3) during the past week, with higher scores indicating more frequent symptoms of depression, and a sum-score of ≥ 16 indicating a risk for clinical depression [28].

In the follow-up, depressive symptoms were reported using the Beck Depression Inventory-II (BDI-II) [29]. This scale comprises 21 four-statement sets (scored from 0 to 3) with each statement reflecting increasing severity of depressive symptoms during the past two weeks [29]. A sum-score of ≥ 14 indicates at least mild depressive symptoms [29].

Both depression scales have good psychometric properties [28–30], and the CES-D has been used extensively and validated also in pregnant populations [30]. We have previously shown that in our sample the CES-D (Cronbach`s α = .88 to .92 in the 14 biweekly measurement points) and the BDI-II (α = .91) showed high internal consistency [16].

### Maternal pre-pregnancy obesity and pregnancy disorders

Maternal pre-pregnancy obesity (BMI, ≥ 30 kg/m^2^), gestational diabetes (yes vs. no) and hypertensive pregnancy disorders (pre-eclampsia, gestational hypertension; yes vs. no) were extracted from the MBR and/or from medical records independently verified by a clinical jury.

### Covariates

These included maternal history of physician-diagnosed depression which was reported in a questionnaire at 12+0–13+6 weeks+days of gestation. Questions on maternal ADHD problems were embedded in the Adult Self-Report (ASR) [31], which the mothers completed in the follow-up (T-score ≥ 65 points indicates borderline significant problems). Maternal age at delivery (years), antidepressant use (yes vs. no), psychotropic medication use (yes vs. no), smoking during pregnancy (did not smoke/ quit during the first trimester/ smoked throughout pregnancy), parity (primiparous vs. multiparous), pre-pregnancy/chronic hypertension (yes vs. no), type 1 diabetes (yes vs. no), child’s sex, gestational length (weeks), birthweight (g) adjusted for sex and gestation length, and family structure (cohabitating/married vs. single) at childbirth were derived from the MBR, and maternal alcohol use (yes vs. no) and education (secondary or less, upper secondary, lower tertiary, upper tertiary) were mother-reported during pregnancy, and child’s age at the follow-up.

### Statistical analyses

We first examined maternal depressive symptoms profiles during pregnancy with a latent profile analysis. We compared solutions with two to eight clusters, and identified the most optimal one by using Akaike Information Criterion, sample size-adjusted Bayesian Information Criterion, and Vuong-Lo-Mendell-Rubin Likelihood Ratio Test and Lo-Mendell-Rubin Adjusted Likelihood Ratio Tests. We then tested if the child ADHD symptom scores, treated as a continuous outcome variable, and the proportion of children with clinically significant ADHD symptoms, treated as a dichotomous variable using the ADHD symptom score 10 or above as a clinical cutoff [27], differed between the groups of mothers with different depressive symptom profiles during pregnancy. These group differences are presented as mean differences (MD) and odds ratios (OR) and their 95% Confidence Intervals (CI) from linear (continuous ADHD symptom scores) and logistic regression analyses (ADHD symptom scores dichotomized at clinical cutoff), respectively.

We also examined if the associations between maternal depressive symptoms during pregnancy and child ADHD symptoms were gestation-week- or trimester-specific. In these tests, we used linear regression analysis when we treated child ADHD symptoms as continuous and logistic regression analysis when we dichotomized child ADHD symptoms scores at the clinical cutoff. Further, in these analyses maternal depressive symptom scores (biweekly values; first trimester value, mean values of the second and third trimester values; trimester-weighted mean value) were square root transformed to improve linear model fitting.

In all of the above analyses we first made adjustments for child’s sex and age at follow-up (model 1). Thereafter, we additionally adjusted for maternal age at childbirth, parity, family structure, education level, type 1 diabetes, chronic hypertension, history of physician-diagnosed depression, antidepressant and other psychotropic medication use, alcohol use and smoking during pregnancy, and gestation length and weight at birth adjusted for sex and gestation length (model 2); for maternal pre-pregnancy obesity, gestational diabetes, gestational hypertension, and pre-eclampsia (model 3); for maternal ADHD problems (model4); and finally, for all of the above and maternal depressive symptoms at follow-up parallel to rating the child (model 5).

We also tested if maternal depressive symptoms after pregnancy added to the prenatal effects with an interaction term of maternal trimester-weighted mean depressive symptoms during pregnancy*maternal depressive symptoms after pregnancy that was added to the linear (continuous ADHD symptom scores) and logistic regression models (ADHD symptoms scores dichotomized at the clinical cutoff). In addition, we tested if maternal depressive symptoms after pregnancy mediated the effects of maternal trimester-weighted mean depressive symptoms during pregnancy using the PROCESS macro for mediation in SPSS 24 with 5000 bootstrapping re-samples with bias-corrected CIs [32,33]. Finally, we conducted sensitivity analyses by running the linear regression analyses separately in groups according to maternal pre-pregnancy obesity and pregnancy disorders, child’s sex, maternal history of physician-diagnosed depression, and maternal ADHD problems. We used MPLUS and SPSS 24 data packages for the analyses.

## Results

Characteristics of the study participants are in S1 Table. Correlations between the covariates with child ADHD symptoms are in S2 Table. Maternal biweekly, trimester-specific and trimester-weighed mean values of depressive symptoms during pregnancy were significantly correlated (Pearson r’s ranged from .51 to .92, all p-values < .001) and were also significantly correlated with depressive symptoms after pregnancy (Pearson r’s ranged from 0.36 to 0.46, all *p*-values < 0.001) [16]. The median number of consecutive depressive symptom measurements during pregnancy in the entire sample was 13 and the interquartile range was 12 to 14. There were altogether 879 (49.4%) women with data on all 14 measurement points during pregnancy and only 334 (18.8%) women had more than two missing values during pregnancy.

### Maternal depressive symptoms during pregnancy and child ADHD symptoms

**Fig 1:**
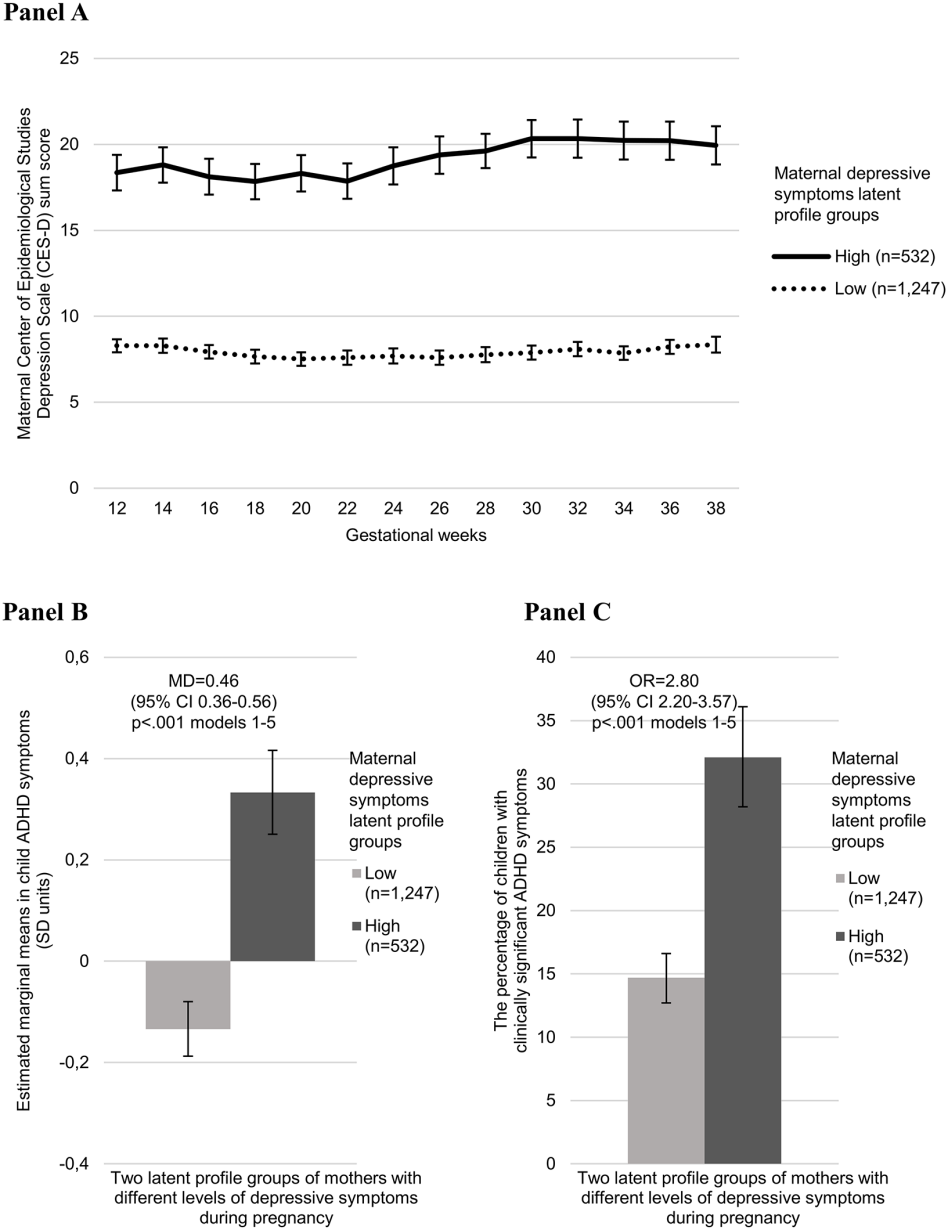
Latent profile analysis showing the most optimal, two group, solution of mothers with consistently low and high scores on the Center of Epidemiological Studies Depression Scale (CES-D) throughout pregnancy (Panel A), and the child’s behavioural symptoms of attention-deficit/hyperactivity disorder on the Conners’ Hyperactivity Index (CHI) estimated marginal mean scores (Panel B), and proportion of children with scores above the clinical cutoff (10) in the CHI (Panel C). Error bars refer to the 95% Confidence Intervals (95% CI), and numbers to mean difference (MD) (Panel B) and odds ratio (OR) (Panel C) and their 95% CIs in model 1, and *p*-values to models 1–5. For different adjustment models, please see footnote in Table 1.

Fig 1 (Panel A) shows that the most optimal latent profile solution (in comparison to solutions with three to eight groups) identified two groups of women with consistently low and high levels of depressive symptoms throughout pregnancy (Akaike Information Criterion = 147821.10, sample-size-adjusted Bayesian Information Criterion = 147920.30, Vuong-Lo-Mendell-Rubin LRT and Lo-Mendell-Rubin-adjusted likelihood ratio test *p*-values < 0.001). For the two latent profile groups, the percentage of women with data on all 14 measurement points compared to the ones with at least one missing value was not significantly different (50.9% in the low and 45.9% in the high depressive symptom level group had all 14 measurement points, *p* = 0.051 for group difference). Fig 1 (Panels B and C) also shows that child ADHD symptoms and the proportion of and odds of children with clinically significant ADHD symptoms were higher in the group of women who had consistently high depressive symptoms throughout pregnancy.

Further, higher maternal biweekly, trimester-specific (S3 Table) and trimester-weighted mean depressive symptom scores (Table 1) were significantly associated with higher ADHD symptom scores and higher odds for clinically significant ADHD symptoms (Table 1) in children. These associations were significant across all adjustment models with all covariates, including maternal ADHD symptoms.

**Table 1:** Association between maternal depressive symptoms during pregnancy and child behavioural symptoms of attention-deficit/hyperactivity disorder on the Conners’ Hyperactivity Index at age 3.5 years.

*Maternal Center of Epidemiological Studies Depression Scale trimester-weighted mean score during pregnancy*	*Child’s Conners’ Hyperactivity Index Sum score*		*Child’s Conners’ Hyperactivity Index Sum score≥10*	
	B (95%CI)	*p*	OR (95%CI)	*p*
Model 1	0.26 (0.22, 0.31)	<0.001	1.83 (1.62, 2.08)	<0.001
Model 2	0.25 (0.21, 0.30)	<0.001	1.85 (1.62, 2.11)	<0.001
Model 3	0.26 (0.21, 0.30)	<0.001	1.86 (1.63, 2.12)	<0.001
Model 4	0.24 (0.20, 0.29)	<0.001	1.77 (1.55, 2.03)	<0.001
Model 5	0.15 (0.10, 0.20)	<0.001	1.49 (1.29, 1.73)	<0.001

B indicates the Standard Deviation (SD) increase in child ADHD symptoms when maternal depressive symptoms increase by 1 SD.

OR (Odds Ratio) indicates the risk of clinically significant ADHD symptoms per 1 SD unit increase in maternal depressive symptoms during pregnancy.

Model 1: adjusted for child sex and age at follow-up

Model 2: adjusted for model 1 + maternal age at childbirth, parity, family structure, education level, type 1 diabetes, pre-pregnancy/chronic hypertension, history of physician-diagnosed depression, antidepressant and other psychotropic medication use, alcohol use and smoking during pregnancy, and gestation length and infant’s birthweight adjusted for sex and gestation length

Model 3: adjusted for model 2 + maternal pre-pregnancy obesity, gestational diabetes, gestational hypertension and pre-eclampsia

Model 4: adjusted for model 3 + maternal ADHD problems

Model 5: adjusted for model 4 + maternal depressive symptoms after pregnancy

### Additive effects of maternal depressive symptoms during and after pregnancy on child ADHD symptoms

**Fig 2:**
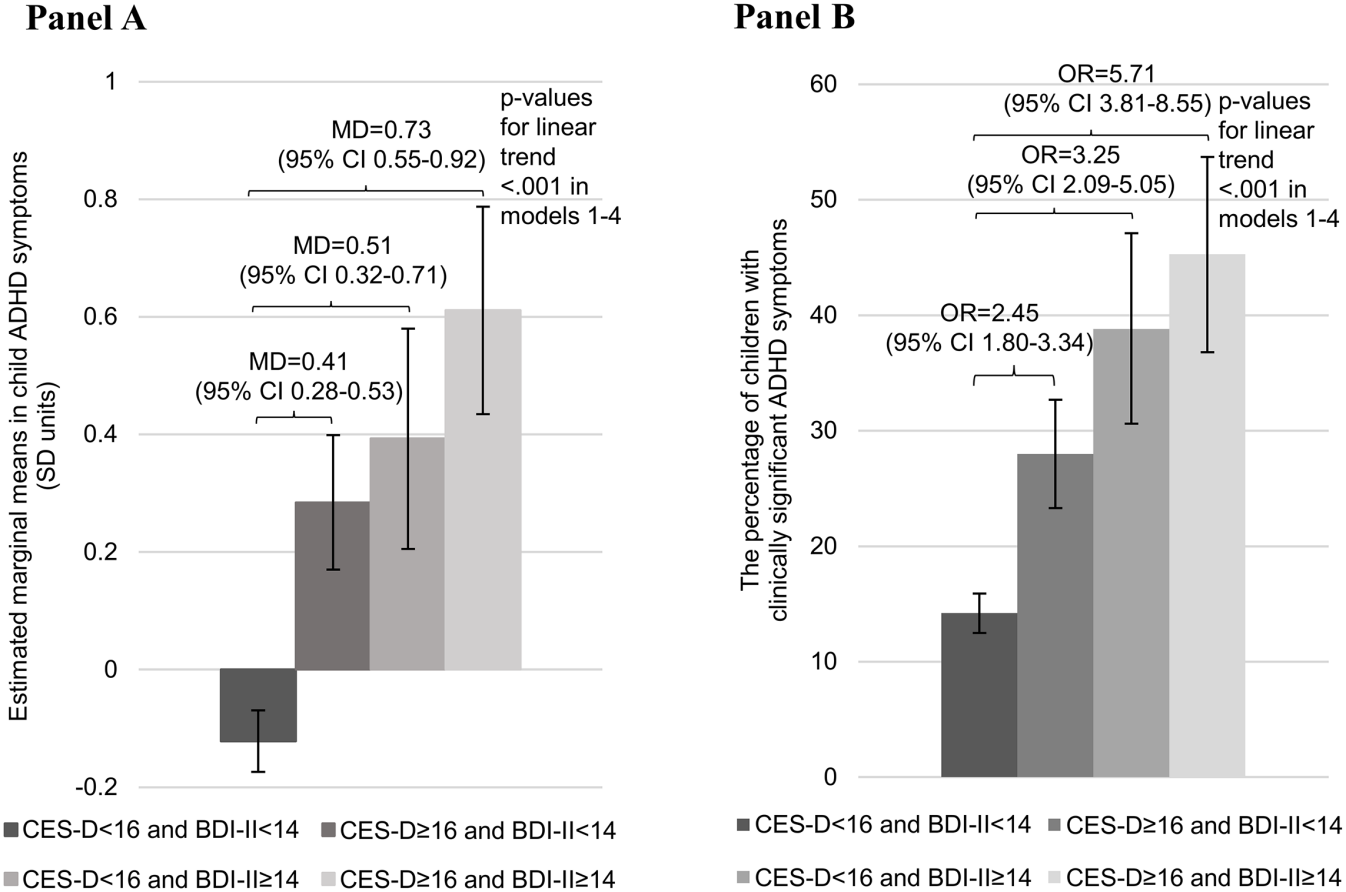
Estimated marginal means (Panel A) of the child’s behavioural symptoms of attention-deficit/hyperactivity disorder on the Conners’ Hyperactivity Index (CHI) and proportion of children with scores above the clinical cutoff (≥10) in the CHI (Panel B) according to the maternal Center of Epidemiological Studies Depression Scale (CES-D) trimester-weighted mean score (≥16) during pregnancy and Beck Depression Inventory-II (BDI-II) sum score (≥14) after pregnancy above and below the clinical cutoffs. Error bars refer to 95% Confidence Intervals (95% CI), and numbers to mean differences (MD) (Panel A) and odds ratios (OR) (Panel B) and their 95% CIs in model 1, and p-values to models 1–4. Women who scored below the clinical cutoff in both the CES-D during pregnancy and in the BDI-II after pregnancy were used as the comparison group. For different adjustment models, please see footnote in Table 1.

Fig 2 (Panels A and B) shows that maternal depressive symptoms after pregnancy added to the effect of maternal depressive symptoms during pregnancy (both *p-*values for interactions = 0.03 for depressive symptoms during pregnancy*depressive symptoms after pregnancy interaction on child continuous and clinically significant ADHD symptoms scores). Across all adjustment models, child ADHD symptom scores and proportion of children with clinically significant symptoms were the highest if the mother reported depressive symptoms above the clinical cutoff both during and after pregnancy (Fig 2).

### Mediation of maternal depressive symptoms during pregnancy on child ADHD symptoms via maternal depressive symptoms after pregnancy

**Fig 3:**
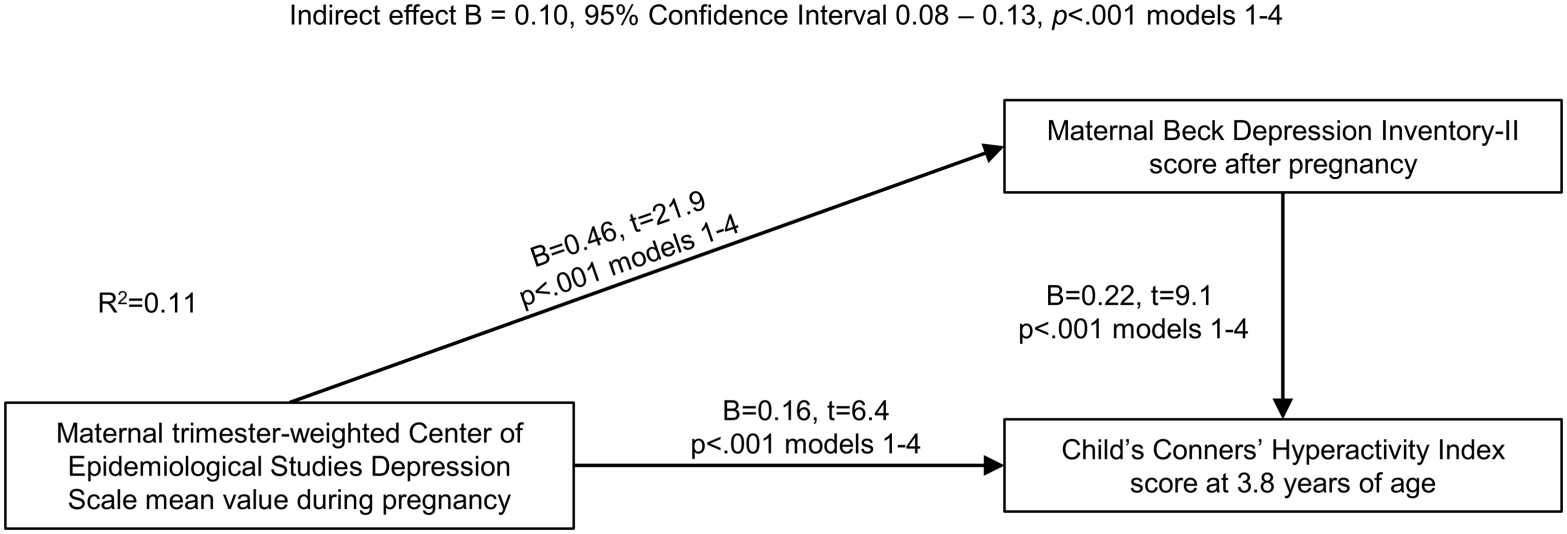
Mediation model of the effects of maternal depressive symptoms during pregnancy on child’s behavioural symptoms of attention-deficit/hyperactivity disorder on the Conners’ Hyperactivity Index via maternal depressive symptoms after pregnancy. Numbers refer to unstandardized regression coefficients (B) and their 95% Confidence Intervals from models adjusted for child’s age and sex, and to the proportion (R^2^) maternal depressive symptoms during and after pregnancy explain of the child’s ADHD symptoms.

Fig 3 shows, that the effect of maternal depressive symptoms during pregnancy on child ADHD symptoms was partially mediated via maternal depressive symptoms after pregnancy. The model also shows that maternal depressive symptoms during pregnancy still had a direct and significant effect on child ADHD symptoms after adjusting for depressive symptoms after pregnancy. Together, depressive symptoms during and after pregnancy accounted for 11.4% of the variation of the child’s ADHD symptoms.

### Sensitivity analyses

S4 Table shows that maternal pre-pregnancy obesity and pregnancy disorders, child’s sex, maternal history of physician-diagnosed depression, or maternal ADHD problems had no effects on the findings: across all groups, the associations between maternal depressive symptoms during pregnancy and child ADHD symptoms were significant.

## Discussion

Our study showed, first, that maternal depressive symptoms throughout pregnancy are associated with child ADHD symptoms. We also showed that maternal depressive symptoms during pregnancy were highly stable, and children of mothers with consistently high depressive symptoms during pregnancy showed higher levels of ADHD symptoms at the age of 3 to 6 years. These children also showed a higher proportion (over 32%) and 2.8-times higher odds for clinically significant ADHD symptoms. It was therefore not surprising that we found no gestation-week or trimester-specific associations between maternal depressive symptoms during pregnancy and child ADHD symptoms. None of these associations were accounted for by a number of perinatal, maternal and neonatal characteristics, and a series of sensitivity analyses demonstrated that the associations did not either vary by maternal pre-pregnancy obesity, hypertensive pregnancy disorders, or gestational diabetes, child’s sex, maternal history of physician-diagnosed depression, or maternal ADHD problems.

Our study also showed that higher levels of maternal depressive symptoms after pregnancy were associated with higher child ADHD symptom scores. These higher levels of depressive symptoms after pregnancy only partially accounted for the prenatal effects as maternal depressive symptoms during pregnancy also had a significant direct effect on the child’s ADHD symptoms when adjusting for the symptoms after pregnancy. They did, however, add to the prenatal effects, such that child ADHD symptom scores and the proportion and odds of children with clinically significant ADHD symptoms were the highest among those women with clinically significant depressive symptoms both during and after pregnancy. Together maternal depressive symptoms during and after pregnancy accounted for 11% of the variation in the child’s ADHD symptoms.

Our findings correspond with the Developmental Origins of Health and Disease (DOHaD) framework suggesting that prenatal exposure to environmental adversity may carry enduring effects on brain developmental sequelae, including risk for ADHD symptomatology [12,13,34]. Our findings are also in alignment with our own recent study on psychiatric behaviour problems [16], and the other two previous prospective studies based on the ALSPAC and the Generation-R cohorts on child attention and hyperactivity problems [19,20] showing that maternal depressive symptoms during pregnancy are associated with child ADHD symptoms. Also in alignment with the ALSPAC study, our study showed that maternal depressive symptoms after pregnancy did not entirely account for the effects of maternal depressive symptoms during pregnancy on child’s ADHD symptoms. In contrast, in the Generation-R study maternal depressive symptoms after pregnancy rendered the prenatal effects on child attention problems non-significant [19]. While our study is to our knowledge the first to formally test for mediation, both the ALSPAC and Generation-R findings point to mediation: the ALSPAC findings point to partial and the Generation-R findings point to full mediation of the prenatal depression effects via the depression effects after pregnancy.

An obvious study limitation is that we are not able to specify the brain structural or functional nor biological or behavioural underlying mechanisms. Existing literature suggests that higher maternal depressive symptoms and/or salivary cortisol levels during pregnancy are linked with altered offspring brain structure and functional connectivity [35], and with cortical thinning in the right hemisphere [36]. Maternal depressive symptoms during pregnancy have also been associated with higher mRNA levels of placental glucocorticoid and mineralocorticoid receptor genes [37], suggesting higher placental glucocorticoid sensitivity. Yet, another study has shown that the risk of borderline clinically significant ADHD problems was over 3-fold in those offspring who were exposed to high levels of maternal licorice consumption during pregnancy [38]. Glycyrrhizin, a natural constituent of licorice, is a potent inhibitor of a placental enzyme protecting the fetus from maternal glucocorticoid excess. Emerging evidence also suggests that inflammatory markers may be involved, as maternal depressive symptoms and pro- and anti-inflammatory cytokines during pregnancy have been shown to be correlated [39]. Clearly, further studies delineating the underlying mechanisms are needed.

Further study limitations relate to child ADHD symptoms being reported by the mother only. However, Leis et al. (2014) found that the effect of maternal prenatal depression on child hyperactivity was significant whether the child was rated by the mother or the teacher. Furthermore, we measured ADHD symptoms dimensionally, and did not use diagnostic criteria, rendering generalizations to ADHD disorder tentative. Since maternal depressive symptoms after pregnancy were self-rated at the time of rating the child’s behaviour, and maternal depression and child behaviour may influence each other, we cannot rule out shared method variance. Sample attrition which was not independent of maternal characteristics also limits the external validity of our findings.

## Conclusions

Our findings show that maternal depressive symptoms during and after pregnancy are associated with child ADHD symptomatology and suggest that early pregnancy screening and preventive interventions focusing on maternal depressive symptoms may benefit not only maternal, but offspring wellbeing. Preventive interventions, suited for pregnancy, are urgently needed, as a recent meta-analysis demonstrated null to very small benefits of existing techniques in decreasing maternal distress during pregnancy [40].

## Supporting information

S1 Table Characteristics of the sample. (DOCX)

S2 Table Correlations and mean differences between maternal and child characteristics used as covariates and the child’s Conners’ Hyperactivity Index sum score (in SD units). (DOCX)

S3 Table Associations (adjusted for child’s sex and age) between maternal biweekly and trimester-specific depressive symptom values during pregnancy and child behavioural symptoms of attention-deficit/hyperactivity disorder on the Conners’ Hyperactivity Index (CHI). (DOCX)

S4 Table Associations between maternal trimester-weighted depressive symptoms mean values during pregnancy and child behavioural symptoms of attention-deficit/hyperactivity disorder on the Conners’ Hyperactivity Index (CHI) according to maternal pregnancy disorders (pre-pregnancy obesity, gestational diabetes, gestational hypertension, pre-eclampsia), history of physician-diagnosed depression and attention deficit hyperactivity disorder problems, and child’s sex. (DOCX)
