# Supplementary material for: Maternal depressive symptoms during and after pregnancy are associated with attention-deficit/hyperactivity disorder symptoms in their 3- to 6-year-old children
Source: PLoS One. 2017 Dec 21;12(12):e0190248. doi: 10.1371/journal.pone.0190248 (PMC5739495; doi:10.1371/journal.pone.0190248)
Supplement: S1 Table — (DOCX) [file pone.0190248.s001.docx]

**S1 Table. Characteristics of the sample.**

| **Maternal characteristics** | **Data available (*n*)** | **Mean (SD)/**  ***n* (%)** |
| --- | --- | --- |
| Age at delivery (years) | 1,779 | 31.9 (4.6) |
| Education | 1,779 |  |
| Primary |  | 36 (2.0 %) |
| Secondary |  | 648 (36.4 %) |
| Tertiary |  | 1,095 (61.6 %) |
| Family structure, single | 1,696 | 25 (1.4 %) |
| Parity, primiparous | 1,774 | 751 (42.2 %) |
| Alcohol use during early pregnancy, yes | 1,763 | 300 (16.9 %) |
| Smoking during pregnancy | 1,779 |  |
| No |  | 1,674 (94.1 %) |
| Quit during the first trimester |  | 59 (3.3 %) |
| Smoked throughout pregnancy |  | 46 (2.6 %) |
| Pre-pregnancy Body Mass Index (kg/m^2^) | 1,779 | 24.4 (4.9) |
| Obese (Body Mass Index≥30) | 1,779 | 239 (13.4 %) |
| Hypertensive pregnancy disorder, yes | 1,779 | 149 (8.4 %) |
| Gestational diabetes, yes | 1,779 | 176 (9.9 %) |
| Chronic hypertension, yes | 1,779 | 71 (4.0 %) |
| Type 1 diabetes, yes | 1,779 | 9 (0.5 %) |
| History of physician-diagnosed depression, yes | 1,689 | 152 (8.5 %) |
| Antidepressant medication use during pregnancy, yes | 1,502 | 40 (2.2 %) |
| Other psychotropic medication use during pregnancy, yes | 1,502 | 11 (0.6 %) |
| Depressive symptoms during pregnancy |  |  |
| Trimester-weighted mean of CES-D sum scores (range 0-60) | 1,779 | 11.3 (6.5) |
| Trimester-weighted mean of CES-D sum scores≥16 | 1,779 | 385 (21.6 %) |
| 1^st^ trimester CES-D sum score (range 0-60) | 1,707 | 11.3 (7.9) |
| 1^st^ trimester CES-D sum score≥16 | 1,707 | 409 (23.0 %) |
| 2^nd^ trimester mean of CES-D sum scores (range 0-60) | 1,774 | 11.0 (6.6) |
| 2^nd^ trimester mean of CES-D sum scores≥16 | 1,774 | 345 (19.4 %) |
| 3^rd^ trimester mean of CES-D sum scores (range 0-60) | 1,732 | 11.7 (7.3) |
| 3^rd^ trimester mean of CES-D sum scores≥16 | 1,732 | 432 (24.3 %) |
| Depressive symptoms after pregnancy |  |  |
| BDI-II sum score (range 0-63) | 1,761 | 6.4 (6.5) |
| BDI-II sum score≥14 | 1,761 | 213 (12.0 %) |
| Maternal self-reported ADHD problems |  |  |
| ADHD problems scale T score≥65 | 1,741 | 85 (4.8 %) |
| **Child characteristics** |  |  |
| Sex, boys | 1,779 | 917 (51.5 %) |
| Gestational age (weeks) | 1,779 | 39.8 (1.6) |
| Birthweight (grams) | 1,779 | 3,524 (523) |
| Age at follow-up (years) | 1,779 | 3.8 (0.5) |
| Child’s behavioural symptoms of attention-deficit/hyperactivity disorder |  |  |
| CHI sum score (range 0-30) | 1,779 | 6.0 (4.6) |
| CHI sum score≥10 | 1,779 | 354 (19.9 %) |

CES-D=Center for Epidemiologic Studies Depression Scale; BDI-II= Beck Depression Inventory –II; ADHD=Attention-deficit/hyperactivity disorder; CHI=Conners’ Hyperactivity Index
