# Supplementary material for: Maternal depressive symptoms during and after pregnancy are associated with attention-deficit/hyperactivity disorder symptoms in their 3- to 6-year-old children
Source: PLoS One. 2017 Dec 21;12(12):e0190248. doi: 10.1371/journal.pone.0190248 (PMC5739495; doi:10.1371/journal.pone.0190248)
Supplement: S2 Table — (DOCX) [file pone.0190248.s002.docx]

**S2 Table.** **Correlations and mean differences between maternal and child characteristics used as covariates and the child’s Conners’ Hyperactivity Index sum score (in SD units).**

| **Maternal characteristics** |  | **Pearson’s r** | **Mean Difference** | ***p*** |
| --- | --- | --- | --- | --- |
| Age at delivery (years) |  | -0.14 |  | <0.001 |
| Education (tertiary=1, primary or secondary=0) |  |  | -0.06 | 0.19 |
| Family structure (married or cohabitating=1, single=0) |  |  | -0.08 | 0.70 |
| Parity (primiparous=1, multiparous=0) |  |  | 0.25 | <0.001 |
| Alcohol use during early pregnancy (yes=1, no=0) |  |  | 0.11 | 0.09 |
| Smoked throughout pregnancy=1, no smoking during pregnancy=0 |  |  | 0.39 | 0.008 |
| Quit smoking during first trimester=1, no smoking during pregnancy=0 |  |  | 0.25 | 0.06 |
| Pre-pregnancy obesity (body mass index ≥ 30=1, body mass index < 30=0) |  |  | -0.00 | 0.97 |
| Hypertensive pregnancy disorder (yes=1, no=0) |  |  | -0.01 | 0.90 |
| Gestational diabetes (yes=1, no=0) |  |  | -0.05 | 0.08 |
| Chronic hypertension (yes=1, no=0) |  |  | -0.07 | 0.55 |
| Type 1 diabetes (yes=1, no=0) |  |  | 0.01 | 0.97 |
| History of physician-diagnosed depression (yes=1, no=0) |  |  | 0.25 | 0.003 |
| Antidepressant use during pregnancy (yes=1, no=0) |  |  | 0.37 | 0.02 |
| Other psychotropic medication use during pregnancy (yes=1, no=0) |  |  | 0.65 | 0.03 |
| Maternal attention deficit/hyperactivity disorder problems (yes=1, no=0) |  |  | 0.54 | <0.001 |
| **Child characteristics** |  |  |  |  |
| Sex (boy=1, girl=0) |  |  | 0.19 | <0.001 |
| Gestational age (weeks) |  | -0.02 |  | 0.36 |
| Birthweight adjusted for sex and gestational age (SD units) |  | -0.06 |  | 0.02 |
| Age at follow-up (years) |  | -0.07 |  | 0.002 |
