# Supplementary material for: Maternal depressive symptoms during and after pregnancy are associated with attention-deficit/hyperactivity disorder symptoms in their 3- to 6-year-old children
Source: PLoS One. 2017 Dec 21;12(12):e0190248. doi: 10.1371/journal.pone.0190248 (PMC5739495; doi:10.1371/journal.pone.0190248)
Supplement: S3 Table — (DOCX) [file pone.0190248.s003.docx]

**S3** **Table.** **Associations (adjusted for child’s sex and age) between maternal biweekly and trimester-specific depressive symptom values during pregnancy and child behavioural symptoms of attention-deficit/hyperactivity disorder on the Conners’ Hyperactivity Index (CHI).**

| ***Maternal Center of Epidemiological Studies Depression Scale biweekly and trimester-specific values during pregnancy*** |  | ***Child’s Conners’ Hyperactivity Index Sum score*** |  |
| --- | --- | --- | --- |
|  | ***n*** | SD unit increase in per 1 SD unit increase (95% CI) | ***p*** |
| **Biweekly values^a^** |  |  |  |
| 12+0-13+6 | 1,707 | 0.21 (0.17, 0.26) | <0.001 |
| 14+0-15+6 | 1,669 | 0.21 (0.16, 0.25) | <0.001 |
| 16+0-17+6 | 1,656 | 0.23 (0.18, 0.27) | <0.001 |
| 18+0-19+6 | 1,658 | 0.21 (0.16, 0.25) | <0.001 |
| 20+0-21+6 | 1,659 | 0.22 (0.17, 0.26) | <0.001 |
| 22+0-23+6 | 1,630 | 0.20 (0.15, 0.24) | <0.001 |
| 24+0-25+6 | 1,613 | 0.21 (0.16, 0.26) | <0.001 |
| 26+0-27+6 | 1,627 | 0.23 (0.18, 0.27) | <0.001 |
| 28+0-29+6 | 1,610 | 0.23 (0.19, 0.28) | <0.001 |
| 30+0-31+6 | 1,614 | 0.23 (0.18, 0.27) | <0.001 |
| 32+0-33+6 | 1,605 | 0.25 (0.21, 0.30) | <0.001 |
| 34+0-35+6 | 1,578 | 0.24 (0.19, 0.28) | <0.001 |
| 36+0-37+6 | 1,517 | 0.21 (0.16, 0.25) | <0.001 |
| 38+0-39+6 | 1,280 | 0.20 (0.15, 0.25) | <0.001 |
| **Trimester-specific values** |  |  |  |
| **1^st^ trimester value** |  |  |  |
| Model 1 | 1,707 | 0.21 (0.17, 0.26) | <0.001 |
| Model 2 | 1,707 | 0.20 (0.15, 0.24) | <0.001 |
| Model 3 | 1,707 | 0.20 (0.15, 0.24) | <0.001 |
| Model 4 | 1,707 | 0.19 (0.14, 0.23) | <0.001 |
| Model 5 | 1,691 | 0.11 (0.06, 0.15) | <0.001 |
| **2^nd^ trimester mean value** |  |  |  |
| Model 1 | 1,774 | 0.25 (0.21, 0.30) | <0.001 |
| Model 2 | 1,774 | 0.24 (0.20, 0.29) | <0.001 |
| Model 3 | 1,774 | 0.24 (0.20, 0.29) | <0.001 |
| Model 4 | 1,774 | 0.23 (0.19, 0.28) | <0.001 |
| Model 5 | 1,756 | 0.14 (0.10, 0.19) | <0.001 |
| **3^rd^ trimester mean value** |  |  |  |
| Model 1 | 1,732 | 0.25 (0.21, 0.30) | <0.001 |
| Model 2 | 1,732 | 0.25 (0.20, 0.29) | <0.001 |
| Model 3 | 1,732 | 0.25 (0.20, 0.29) | <0.001 |
| Model 4 | 1,732 | 0.23 (0.19, 0.28) | <0.001 |
| Model 5 | 1,716 | 0.15 (0.10, 0.20) | <0.001 |

^a^ Analyses were adjusted for child sex and age at follow-up. After additional adjustments for maternal age at childbirth, parity, family structure, education level, type 1 diabetes, chronic hypertension, history of physician-diagnosed depression, antidepressant and other psychotropic medication use, alcohol use and smoking during pregnancy, and gestation length and infant’s birthweight adjusted for sex and gestation length, maternal pregnancy disorders, maternal ADHD problems, and maternal depressive symptoms at follow-up parallel to rating the child all *p*-values remained <.001.

Model 1: adjusted for child sex and age at follow-up

Model 2: adjusted for model 1 + maternal age at childbirth, parity, family structure, education level, type 1 diabetes, chronic hypertension, history of physician-diagnosed depression, antidepressant and other psychotropic medication use, alcohol use and smoking during pregnancy, and gestation length and infant’s birthweight adjusted for sex and gestation length

Model 3: adjusted for model 2 + maternal pre-pregnancy obesity, gestational diabetes, gestational hypertension and pre-eclampsia

Model 4: adjusted for model 3 + maternal ADHD problems

Model 5: adjusted for model 4 + maternal depressive symptoms after pregnancy
