# Supplementary material for: Maternal depressive symptoms during and after pregnancy are associated with attention-deficit/hyperactivity disorder symptoms in their 3- to 6-year-old children
Source: PLoS One. 2017 Dec 21;12(12):e0190248. doi: 10.1371/journal.pone.0190248 (PMC5739495; doi:10.1371/journal.pone.0190248)
Supplement: S4 Table — (DOCX) [file pone.0190248.s004.docx]

**S4 Table.** **Associations between maternal trimester-weighted depressive symptoms mean values during pregnancy and child behavioural symptoms of attention-deficit/hyperactivity disorder on the Conners’ Hyperactivity Index (CHI) according to maternal pregnancy disorders (pre-pregnancy obesity, gestational diabetes, gestational hypertension, pre-eclampsia), history of physician-diagnosed depression and attention deficit hyperactivity disorder problems, and child’s sex.**

| **Maternal trimester-weighted Center of Epidemiological Studies Depression Scale mean score in standard deviation units** | **Child’s Conners’ Hyperactivity Index sum score in standard deviation units** | | | |  |
| --- | --- | --- | --- | --- | --- |
|  | SD unit increase per 1 SD unit increase (95% CI) | ***p*** | SD unit increase per 1 SD unit increase (95% CI) | ***p*** | |
| **Maternal characteristics** |  |  |  |  | |
| **Pregnancy disorders** | **yes n=436** |  | **no n*=*1,343** |  | |
| Model 1 | 0.28 (0.19, 0.37) | 0.002 | 0.26 (0.21, 0.31) | <0.001 | |
| **History of physician-diagnosed depression** | **yes n=151** |  | **no n=1,533** |  | |
| Model 1 | 0.23 (0.08, 0.38) | 0.002 | 0.27 (0.23, 0.32) | <0.001 | |
| **Attention deficit hyperactivity disorder problems** | **yes n=65** |  | **no n=1,264** |  | |
| Model 1 | 0.33 (0.09, 0.58) | 0.009 | 0.25 (0.20, 0.29) | <0.001 | |
| **Child characteristics** |  |  |  |  | |
| **Sex** | **boys n=917** |  | **girls n=862** |  | |
| Model 1^a^ | 0.30 (0.24, 0.36) | <0.001 | 0.23 (0.16, 0.29) | <0.001 | |

Model 1: adjusted for child sex and age at follow-up

^a^ Model not adjusted for child sex
